# Lumbar Puncture

**Published:** 1899-08-12

**Authors:** J. O. Symes

**Affiliations:** Bacteriologist, Bristol Royal Infirmary


### Hospital Clinics and Medical Progress.

### LUMBAR PUNCTURE.

By J. O. Symes, M.D., Bacteriologist, Bristol Royal Infirmary.

Withdrawal of cerebro-spinal fluid may be practised either for purposes of diagnosis or treatment. The therapeutic results following the reduction of pressure by this means have not been encouraging, but much light may be thrown upon the case by the chemical, bacteriological, and microscopic examination of the fluid obtained. Lumbar puncture is then chiefly resorted to with a view to assisting diagnosis. The technique of the operation is as follows: The patient, if not anæsthetised, is seated in a chair, and the body is strongly bent forward so as to make as great a space as possible between the spines and laminæ of the lumbar vertebræ. The skin over the lumbar vertebræ is then disinfected, being washed with ether and then with a solution of 1 in 500 perchloride of mercury. A fine cannula and trocar, previously sterilised by boiling, are then thrust into the spinal cavity well below the termination of the cord. The puncture should be made between the second and third, third and fourth, or fourth and fifth lumbar spines, or at the level of a line joining the highest points of the iliac crests. In children the trocar may be placed in the middle line immediately between two spines, but in adults it is preferable to select a spot a quarter to half an inch from the middle line, opposite the junction of the middle with the lower third of a spinous process. The point of the trocar should be directed upwards and inwards, and the dura mater will be pierced one to three inches from the surface. When the trocar is withdrawn the amount of fluid that will escape through the cannula will vary according to the nature of the disease; thus in meningitis only a few drops may be secured, whilst in hydrocephalus it may amount to one or two ounces or more. In children and in persons unlikely to stand the pain of the initial puncture it is best to employ an anæsthetic, the patient being kept lying on the side, with the back well arched. With a trocar the operation is generally not more painful than paracentesis thoracis, but the rapid drawing off of fluid with an aspirator sometimes gives rise to much pain, and for this reason the use of this instrument has been abandoned. The wound caused by the trocar is afterwards dressed with gauze soaked in collodion.

Occasionally severe pains extending to the lower limbs result from lumbar puncture, and the rapid removal of the fluid in a case of cerebral tumour has been followed by sudden death. As a therapeutic measure this procedure has been chiefly used for the relief of tension in cases of hydrocephalus, cerebral tumour, and spinal hæmorrhage. It has also been recommended in acute mania, and in some forms of chlorosis. The results in these cases already recorded are not encouraging.

For purposes of diagnosis the fluid should be examined for albumen, blood, cells, and micro-organisms, and a note should also be made of the quantity, reaction, colour, and specific gravity. The quantity of albumen serves to distinguish between a simple hydrocephalus and an inflammatory effusion. In the former not more than a half per cent. is usually present; in the latter, one per cent. is a fair average.

Inflammatory effusion, such as is found in meningitis, is distinguished, too, by its cloudiness, its coagulability, and by the fact that it will usually be found to contain cells. The presence of blood would point to rupture of a spinal or cerebral vessel, though it is obvious that here error might arise from accidental injury to vessels by the exploring needle.

The most useful results have undoubtedly been obtained by bacteriological examination of the fluid, it being possible by this means to distinguish between a meningitis of tubercular origin, and the same affection due to other micro-organisms. Thus Furbinger (Berlin *Klin. Woch.*, April, 1898) found tubercle bacilli in 27 out of 86 cases investigated, and verified the diagnosis by post-mortem examination. In three other cases which terminated fatally, and in which tubercle bacilli were found, no post-mortem was held. One case of spinal suppuration showed pneumococcus, and a second the micrococcus of cerebro-spinal meningitis. Seven cases in which the bacteriological examination was negative proved on post-mortem examination to be tuberculous. Osler reports 21 cases of cerebro-spinal fever, in 16 of which a diagnosis was made by the aid of this procedure (*British Medical Journal*, June 24th, 1899). Jacoby, who has had a wide experience of lumbar puncture, reports an interesting case in which by this means it was possible in a case of middle ear suppuration to diagnose a secondary streptococcal cerebro-meningitis (New York *Medical Journal*, December 28th, 1895). When the fluid drawn off is purulent it is frequently possible to demonstrate the organism exciting the disease by growth on culture media or by means of coverslip preparations. In suspected tubercular cases, and always when the fluid is cloudy, it is best to receive it in a sterile vessel and reserve it for centrifugalisation and inoculation. It has been proposed that lumbar puncture should be utilised for the purpose of applying medicinal substances to the membranes of the cord in diseased conditions, but we are not aware that this suggestion has been adopted. The advantages of diminished tension obtained by puncture are, as we have previously stated, said to be of no practical value, except perhaps in cases of spinal hæmorrhage, and it is at the present time solely as an aid to diagnosis that the operation survives. The technique is by no means difficult, especially in children, and may readily be obtained by practice in the post-mortem room.

The danger to the patient is small, probably not greater than that of aspiration of a pleural effusion. Pain is seldom experienced if the fluid be drawn off slowly, and although death has ensued immediately after the procedure, it must be remembered that in the conditions in which this has occurred, viz., cerebral tumour or abscess, a sudden fatal termination is not uncommon.

